# Development and validation of a difficult laryngoscopy prediction model using machine learning of neck circumference and thyromental height

**DOI:** 10.1186/s12871-021-01343-4

**Published:** 2021-04-21

**Authors:** Jong Ho Kim, Haewon Kim, Ji Su Jang, Sung Mi Hwang, So Young Lim, Jae Jun Lee, Young Suk Kwon

**Affiliations:** 1 Department of Anesthesiology and Pain Medicine, Chuncheon Sacred Heart Hospital, 77 Sakju-ro, Chuncheon, 24253 South Korea; 2 Institute of New Frontier Research Team, Hallym University, Chuncheon, South Korea

**Keywords:** Machine learning, Difficult laryngoscopy, Thyromental height, Neck circumference

## Abstract

**Background:**

Predicting difficult airway is challengeable in patients with limited airway evaluation. The aim of this study is to develop and validate a model that predicts difficult laryngoscopy by machine learning of neck circumference and thyromental height as predictors that can be used even for patients with limited airway evaluation.

**Methods:**

Variables for prediction of difficulty laryngoscopy included age, sex, height, weight, body mass index, neck circumference, and thyromental distance. Difficult laryngoscopy was defined as Grade 3 and 4 by the Cormack-Lehane classification. The preanesthesia and anesthesia data of 1677 patients who had undergone general anesthesia at a single center were collected. The data set was randomly stratified into a training set (80%) and a test set (20%), with equal distribution of difficulty laryngoscopy. The training data sets were trained with five algorithms (logistic regression, multilayer perceptron, random forest, extreme gradient boosting, and light gradient boosting machine). The prediction models were validated through a test set.

**Results:**

The model’s performance using random forest was best (area under receiver operating characteristic curve = 0.79 [95% confidence interval: 0.72–0.86], area under precision-recall curve = 0.32 [95% confidence interval: 0.27–0.37]).

**Conclusions:**

Machine learning can predict difficult laryngoscopy through a combination of several predictors including neck circumference and thyromental height. The performance of the model can be improved with more data, a new variable and combination of models.

**Supplementary Information:**

The online version contains supplementary material available at 10.1186/s12871-021-01343-4.

## Background

The difficult airway is challenging for ventilation by facemask or a supraglottic airway, laryngoscopy, and/or intubation and poses difficulty in securing an emergency surgical airway. Difficult laryngoscopy (DL) was defined as the inability to visualize parts of the vocal cords after several conventional laryngoscopy attempts by a trained anesthesiologist [1]. Although video laryngoscopes are widely used in difficult airway management, there are cases where a video laryngoscope cannot be used, and intubation of the trachea may fail even if the larynx is visible [2, 3]. When there is active bleeding or vomitus in the oral cavity or around the laryngopharynx area, it may be difficult to use a video laryngoscope. Direct laryngoscopy technique is a basic and important technique for tracheal intubation.

Various methods of predicting difficult airway have been reported when direct laryngoscopy technique was used [4–9]. However, there are limited methods for evaluating the airway in unconscious patients, patients with difficult communication, or patients with limited movement of the neck and mouth. Neck circumference (NC) and thyromental height (TMHT) can be measured regardless of the patient’s ability to communicate and move neck and mouth. This study aims to evaluate DL using NC and TMHT and develop and validate a prediction model using machine learning rather than conventional methods.

## Materials and methods

This study was conducted after approval by the Institutional Review Board / Ethics Committee of Chuncheon Sacred Heart Hospital, Hallym University (IRB No. 2020–09-011), All authors have confirmed the research guidelines and regulations of the committee that approved the study, and all studies have been conducted in accordance with the relevant guidelines and regulations. This study did not include vulnerable participants, including under 18 years of age, and informed consent was obtained from all subjects. The data of patients who had undergone general anesthesia at Hallym University Chuncheon Sacred Heart Hospital between January 18, 2019, and September 25, 2020, were collected from preanesthesia and anesthesia records.

Exclusion criteria are as follows:

- Under 18 years old
- Regional anesthesia
- Major external facial or neck abnormalities
- Laryngeal abnormalities or tumors
- Laryngeal mask used
- Mask ventilation only
- Video laryngoscope used
- Fiberoptic scope used
- Missing data
- Endotracheal intubation or tracheostomy stated before anesthesia

### Predictors of difficult laryngoscopy

DL prediction included age, sex, height, weight, body mass index, NC, and TMHT. NC was defined as the circumference at the level of the thyroid cartilage [8]. TMHT was defined as the height between the anterior border of the thyroid cartilage (on the thyroid notch just between the two thyroid laminae) and the anterior border of the mentum (on the mental protuberance of the mandible), with the patient lying supine with her/his mouth closed [4].

### Intubation and difficult laryngoscopy

Tracheal intubation procedures were performed through a standardized method by seven attending anesthesiologists and five resident anesthesiologists. Standard Macintosh metallic single-use disposable laryngoscope blades (INT; Intubrite Llc, Vista, CA, USA) were used. Direct laryngoscopy views were classified following the Cormack-Lehane grades: Grade 1 = most of the glottic opening is visible; Grade 2 = only the posterior portion of the glottis or only arytenoid cartilages are visible; Grade 3 = only the epiglottis but no part of the glottis is visible; Grade 4 = neither the glottis nor the epiglottis is visible. Cormack-Lehane 3 and 4 indicated DL and were combined into the difficult class. Cormack-Lehane 1 and 2 were combined into the non-difficult laryngoscopy (NDL) class.

### Machine learning and statistics

The dataset was created with the result of DL and the factors for its prediction. The dataset was randomly divided into a training set (80%) and a test set (20%), but each dataset had the same NDL and DL class ratio. A prediction model was created through the training set with a machine learning algorithm. The prediction model was validated through the test set. In general, since the DL class is much smaller than the NDL class, there is an imbalance of training data. In this study, DL class oversampling was used through a synthetic minority oversampling technique (SMOTE) [10] to solve the data imbalance problem. The parameters used in SMOTE and algorithms are summarized in supplementary Table 1.

The training set was normalized by Min-Max scaling after applying SMOTE. The test set was normalized according to the Min-Max scaling of the training set. All training sets were trained with five algorithms. The algorithms included logistic regression (LR), multilayer perceptron (MLP), BRF, extreme gradient boosting (XGB), and light gradient boosting machines (LGBM) [11–14]. The predictive models learned with five algorithms were validated through the test set. Because the dataset is unbalanced, each model’s validation results were evaluated by the area under the curve of the receiver operating characteristic curve (AUROC) and the area under the curve of the precision-recall curve (AUPRC) [15]. The threshold with the optimal balance between false positive and true positive rates was determined as maximum geometric mean of sensitivity (recall) and specificity. The sensitivity, specificity, recall and accuracy were calculated at the determined threshold. The confidence interval (CI) was calculated as follows:

$$ CI=\overline{x}\pm Z\frac{s}{\sqrt{n}} $$

($\overline{x}$: mean, *Z*: Z value (1.96 at 95%), *s*: standard deviation, *n*: number of observation)

Developing and validating all models were processed by Anaconda (Python version 3.7, https://www.anaconda.com; Anaconda Inc., Austin, TX, USA), the XGBoost package version 0.90 (https://xgboost.readthedocs.io), the LGBM package version 2.2.3 (https://lightgbm.readthedocs.io/en/latest/Python-Intro.html), and the imbalanced-learn package version 0.5.0 (SMOTE, BRF; https://imbalanced-learn.readthedocs.io), scikit-learn 0.24.1(MLP, LR; https://scikit-learn.org/stable/index.html). The data set factors were analyzed by SPSS (IBM Corporation, Armonk, NY, USA). Continuous data are expressed with the median and interquartile range, and categorical data are expressed as number and percentage. Continuous predictors were compared with the Mann-Whitney test and categorical predictors by the chi-squared test. All *P*-values were two-sided, and a P-value < 0.05 was considered indicative of statistical significance.

## Results

From January 18, 2019 to September 25, 2020, 7765 patients underwent surgery under general anesthesia and tracheal intubation, excluding local anesthesia, and 1677 patients were eligible in the study. The predictors of DL are summarized in Table 1. Altogether 1467 patients had NDL, and 210 patients had DL. Age, male, TMHT, and NC had significant differences between the NDL and DL groups. The train dataset included 1341 patients (NDL: 1173, DL: 168) and the test dataset included 336 patients (NDL: 294, DL: 42).

**Table 1:** The predictors of difficult laryngoscopy in the dataset

	No difficult laryngoscopy (*n* = 1467)	Difficult laryngoscopy (*n* = 210)	P
Age, median (IQR), years	57 (43, 66)	61 (49, 68)	0.004
Male, number (%)	793 (54.1)	132 (62.9)	0.016
Height, median (IQR), cm	162.7 (156.1, 169.7)	164.9 (157.0, 170.1)	0.162
Weight, median (IQR), kg	66.5 (57.8, 75.0)	66.8 (59.0, 78.8)	0.264
Body mass index, median (IQR), kg/m^2^	24.9 (22.8, 27.4)	25.1 (23.2, 27.3)	0.425
Thyromental distance, median (IQR), cm	5.5 (4.7, 6.4)	5.4 (4.4, 6.2)	0.032
Neck circumference, median (IQR), cm	36.8 (34.1, 39.2)	37.8 (35.4, 40.0)	0.001

*IQR* interquartile range

**Fig. 1:**
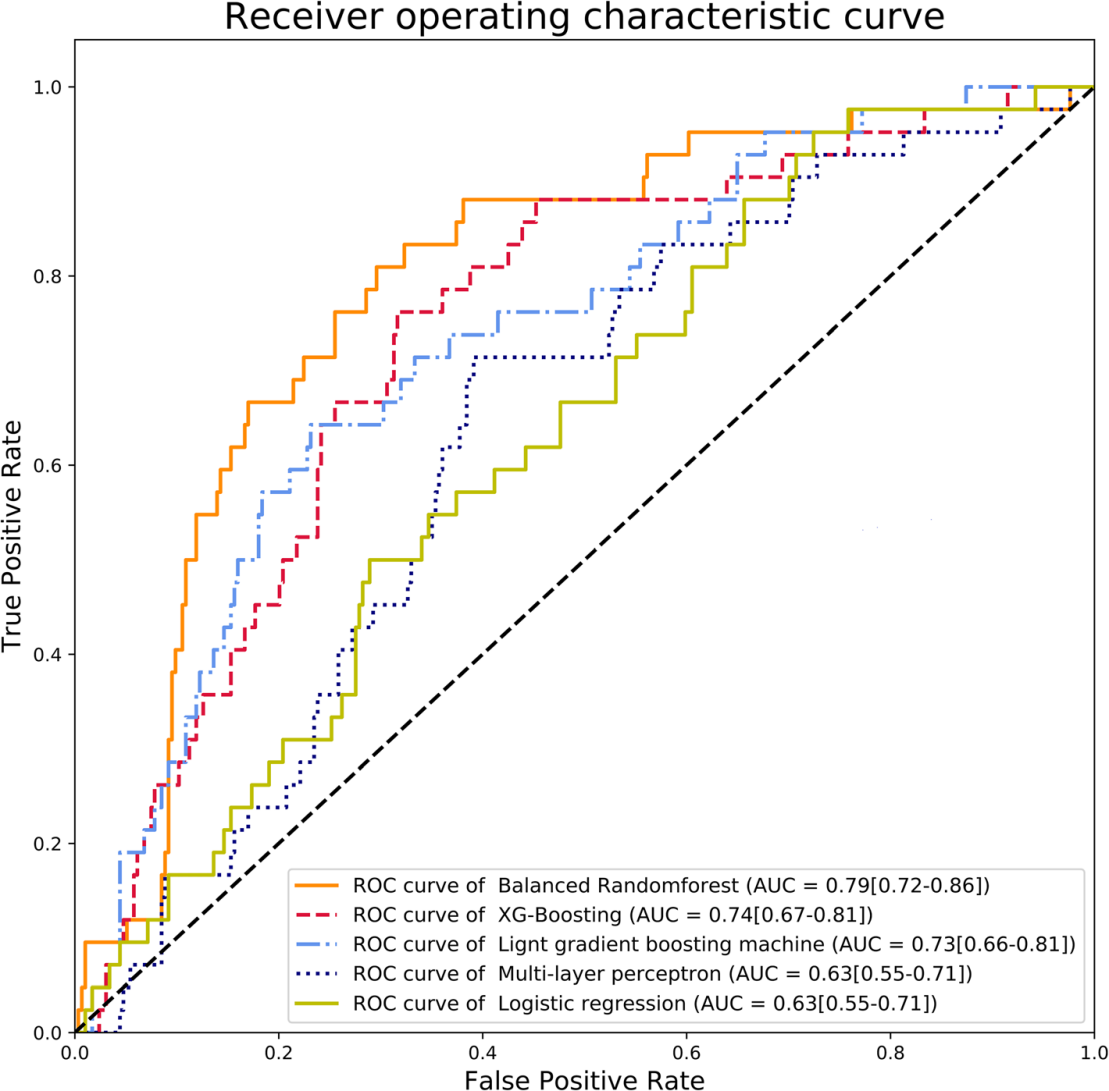
The area under the receiver operating characteristic curve of the machine learning models for difficult laryngoscopy in the test set. AUC (area under curve [95% confidence interval])

**Fig. 2:**
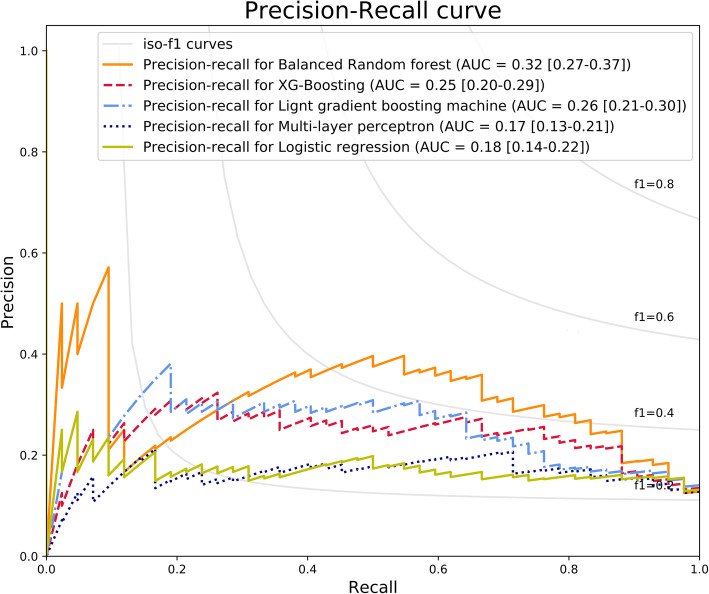
The area under the precision-recall curve of the machine learning models for difficult laryngoscopy in the test set. AUC (area under curve [95% confidence interval])

The AUROC (95% confidence interval [CI]) of TMHT and NC as a single predictor before dividing into training set and test set were 0.45 (0.41–0.50) and 0.57 (0.53–0.61), respectively. The AUROCs showing the performance of the machine learning model for DL prediction are presented in Fig. 1**.** In the evaluation of the model through the receiver operating characteristic curve, the model using the BRF algorithm showed the best performance with AUROC (95% CI) of 0.79 (0.72–0.86), and the model using MLP and LR showed the worst performance with AUROC (95% CI) of 0.63 (0.55–0.71). The AUPRCs showing the performance of the machine learning model for DL prediction are presented in Fig. 2**.** In the evaluation of the model through the precision-recall curve, the model using the BRF algorithm showed the best performance with AUPRC (95% CI) of 0.32 (0.27–0.37), and the model using MLP showed the worst performance with AUPRC (95% CI) of 0.17 (0.13–0.21). The sensitivity, specificity, and accuracy of the DL prediction models are summarized in Table 2. The BRF model had the highest sensitivity (90%), and the LGBM model had the highest specificity (91%) and accuracy (83%).

**Table 2:** Sensitivity (recall) and specificity and accuracy according to difficult laryngoscopy prediction model

	Threshold	Sensitivity (95CI)	Specificity (95CI)	Presision (95CI)	Accuracy (95CI)
BRF	0.54	0.90 (0.88–0.92)	0.58 (0.55–0.61)	0.23 (0.21–0.25)	60% (57–63)
XGB	0.23	0.54 (0.51–0.57)	0.78 (0.76–0.80)	0.25 (0.23–0.27)	69% (66–72)
LGBM	0.36	0.22 (0.20–0.24	0.91 (0.89–0.93)	0.28 (0.26–0.30)	83% (81–85)
MLP	0.37	0.49 (0.46–0.52)	0.60 (0.57–0.63)	0.19 (0.17–0.21)	61% (58–64)
LR	0.36	0.66 (0.63–0.69)	0.56 (0.53–0.59)	0.17 (0.15–0.19)	57% (54–60)

*95CI* 95% confidence interval, *BRF* balanced random forest, *XGB* extreme gradient boosting, *LGBM* light gradient boosting machines, *MLP* multilayer perceptron, *LR* logistic regression

## Discussion

TMHT and NC did not show good results as single predictors of DL. Five machine learning algorithms (BRF, XGB, LGBM, MLP, LR) were applied to predict DL using seven predictors, including TMHT and NC, which can be measured even in limited airway assessment. AUROC and AUPRC, which evaluate the model’s performance, showed the best performance in the model to which BRF was applied but did not show excellent performance. Sensitivity was highest in the model to which BRF was applied. Specificity and accuracy were the highest in the model to which LGBM was applied.

In many studies, the NC has been associated with difficult airway intubation in obese patients [8, 16, 17]. Thyromental height has also been reported as a predictor of difficult airway management [4, 16–20]. These findings support that the NC and TMHT may be predictors of DL. Several studies showed promising results, even with a single predictor [4, 16–22]. However, the previous studies are different from those of ours. The vast majority of the studies on prediction of difficult airway using NC is on obese patients so data in non-obese are insufficient [8, 16, 17]. There were also differences in the primary outcome (difficult intubation vs. DL) [8, 18, 20–22]. There may be differences in some TMHT studies because the patient population is of different races from the patient population in our study. Some studies have targeted specific patient populations such as coronary bypass patients, elderly and endotracheal intubation double-lumen tubes [16, 18, 20]. In some TMHT studies, like ours, the primary outcome was DL. In their study, TMHT as a predictor showed excellent performance in predicting DL [4, 17]. However, it is difficult to generalize because they were not a large-scale study and conducted for a specific race. In clinical practice, it is difficult to predict DL with a single predictor, including TMHT. Numerous studies have reported methods of predicting difficult airway, but no reliable way of predicting difficult airway exists yet [23–26]. Using multiple tests to predict difficulty in airway management may be a better predictor than any single test used in isolation [27].

Machine learning is being used to analyze the importance of clinical parameters and their combinations for prognosis, e.g. prediction of disease progression, extraction of medical knowledge for outcome research, therapy planning and support, and overall patient management [28]. Therefore, it may be necessary to apply machine learning even in difficult airway predictions. The models that predict difficult airways using machine learning has been reported in a few studies [29, 30]. Langerson and colleagues showed that the computer-based boosting method is superior to other conventional methods in predicting difficult tracheal intubation. Their results show that machine learning can be effective in predicting difficult airways. However, the predictors used by them included body mass index, age, Mallampati class, thyromental distance, mouth opening, macroglossia, sex, receding mandible, and snoring, so it cannot be applied to patients with limited airway assessment as in our study [30]. Moustafa and colleagues also reported a method of predicting DL using machine learning, as in our study. They used nine predictors and showed an AUROC of 0.79, which is the same as our study results. However, it is difficult to compare the model’s performance with our products because their results are the results of training with only 100 patients and do not include the model’s validation results through the test set. In addition, since predictors include interincisor distance, thyromental distance, sternomental distance, modified Mallampati score, upper lip bite test, and joint extension, it cannot be applied to patients with limited airway evaluation [29].

This study’s strength is that machine learning algorithms were used in the development of models to predict DL, and the models were validated through a test set. However, there are some limitations to this study. First, the model for predicting DL developed in this study does not show excellent performance with AUROC and especially AUPRC. Moreover, there is no predictive model with high sensitivity, high specificity, and accuracy. We did not calculate the number of samples required for the study. When applying machine learning algorithms, a lot of data is required. Often more data is required than is reasonably required by classical statistics. In particular, nonlinear models require as much data as possible. As few as thousands to tens of thousands of samples may be required [31]. In this study, unlike previous study with same algorithms [32], it was conducted prospectively, and we tried to include the maximum amount of training data in consideration of the expected study period and the difficulty of obtaining data. After oversampling with SMOTE, each class of train set was 1173. However, to improve the performance of a predictive model, the model needs to learn more data [33]. Second, the data used to train and validate the model can be difficult to apply to pediatric patients or other races because the data population is adults and mostly Koreans. Asian populations have statistically different dimensions from Caucasian populations in terms of chin arch, face length, and nose protrusion.

## Conclusions

In this study, NC and TMHT, which can be used even in patients with limited airway evaluation, were used as predictors of DL. Data were learned through five machine learning algorithms to develop a DL prediction model, and the prediction model was validated. The overall model performance was not excellent, but some predictive models showed high sensitivity, specificity, or accuracy, depending on the model. More data can be trained or new predictors can be added to increase performance. To overcome each model’s weaknesses, a method of applying an ensemble of a model with high sensitivity and a model with high specificity can be considered.

## Supplementary Information

**Additional file 1: Supplementary table 1.** The parameters used in SMOTE and algorithms.

## Data Availability

The datasets used and/or analyzed during the current study are available from the corresponding author on reasonable request.

## Abbreviations

DL: Difficult laryngoscopy
NC: Neck circumference
TMHT: Thyromental height
NDL: Non-difficult laryngoscopy
LR: Logistic regression
MLP: Multilayer perceptron
BRF: Balanced random forest
XGB: Extreme gradient boosting
LGBM: Light gradient boosting machine
AUROC: Area under receiver operating characteristic curve
AUPRC: Area under the curve of the precision-recall curve
CI: Confidence interval
