# Supplementary material for: Development and validation of a difficult laryngoscopy prediction model using machine learning of neck circumference and thyromental height
Source: BMC Anesthesiol. 2021 Apr 21;21:125. doi: 10.1186/s12871-021-01343-4 (PMC8059322; doi:10.1186/s12871-021-01343-4)
Supplement: Supplementary file 1 — Additional file 1: Supplementary table 1. The parameters used in SMOTE and algorithms. [file 12871_2021_1343_MOESM1_ESM.docx]

Supplementary table 1. The parameters used in SMOTE and algorithms

| *SMOTENC(categorical_features=[1], k_neighbors=5, n_jobs=None, sampling_strategy='auto')* |
| --- |
| *BalancedRandomForestClassifier(bootstrap=True, ccp_alpha=0.0, class_weight=None,*  *criterion='gini', max_depth=None,*  *max_features='auto', max_leaf_nodes=None,*  *max_samples=None, min_impurity_decrease=0.0,*  *min_samples_leaf=2, min_samples_split=2,*  *min_weight_fraction_leaf=0.0, n_estimators=100,*  *n_jobs=None, oob_score=False,*  *random_state=328482, replacement=False,*  *sampling_strategy='auto', verbose=0,*  *warm_start=False)* |
| *XGBClassifier(base_score=0.5, booster='gbtree', colsample_bylevel=1,*  *colsample_bynode=1, colsample_bytree=1, gamma=0,*  *learning_rate=0.1, max_delta_step=0, max_depth=3,*  *min_child_weight=1, missing=None, n_estimators=100, n_jobs=1,*  *nthread=None, objective='binary:logistic', random_state=42,*  *reg_alpha=0, reg_lambda=1, scale_pos_weight=1, seed=None,*  *silent=None, subsample=1, verbosity=1)* |
| *LGBMClassifier(boosting_type='gbdt', class_weight=None, colsample_bytree=1.0,*  *importance_type='split', learning_rate=0.1, max_depth=-1,*  *min_child_samples=20, min_child_weight=0.001, min_split_gain=0.0,*  *n_estimators=100, n_jobs=-1, num_leaves=31, objective='binary',*  *random_state=None, reg_alpha=0.0, reg_lambda=0.0, silent=True,*  *subsample=1.0, subsample_for_bin=200000, subsample_freq=0)* |
| *MLPClassifier(activation='relu', alpha=0.0001, batch_size='auto', beta_1=0.9,*  *beta_2=0.999, early_stopping=False, epsilon=1e-08,*  *hidden_layer_sizes=(100,), learning_rate='constant',*  *learning_rate_init=0.001, max_fun=15000, max_iter=200,*  *momentum=0.9, n_iter_no_change=10, nesterovs_momentum=True,*  *power_t=0.5, random_state=0, shuffle=True, solver='lbfgs',*  *tol=0.0001, validation_fraction=0.1, verbose=False,*  *warm_start=False)* |
| *LogisticRegression(C=1.0, class_weight=None, dual=False, fit_intercept=True,*  *intercept_scaling=1, l1_ratio=None, max_iter=100,*  *multi_class='auto', n_jobs=None, penalty='l2',*  *random_state=0, solver='lbfgs', tol=0.0001, verbose=0,*  *warm_start=False)* |
